# Evaluating tooth strontium and barium as indicators of weaning age in Pacific walruses

**DOI:** 10.1111/2041-210X.13482

**Published:** 2020-09-28

**Authors:** Casey T. Clark, Lara Horstmann, Nicole Misarti

**Affiliations:** ^1^ Joint Institute for the Study of Atmosphere and Ocean University of Washington Seattle WA USA; ^2^ Cooperative Institute for Climate, Ocean, and Ecosystem Studies University of Washington Seattle WA USA; ^3^ College of Fisheries and Ocean Sciences University of Alaska Fairbanks Fairbanks AK USA; ^4^ Water and Environmental Research Center University of Alaska Fairbanks Fairbanks AK USA

**Keywords:** lactation, LA‐ICP‐MS, marine mammal, nursing, pinniped, trace elements

## Abstract

1. Lactation length and weaning age provide important information about maternal investment, which can reflect the health and nutritional status of the mother, as well as broader reproductive strategies in mammals. Calcium‐normalized strontium (Sr) and barium (Ba) concentrations in the growth layers of mammalian teeth differ for nursing animals and those consuming non‐milk foods, thus can be used to estimate age‐at‐weaning. To date, this approach has been used only for terrestrial animals, and almost exclusively for primates.
2. The goal of this study was to determine whether Sr and Ba concentrations in the cementum of Pacific walrus *Odobenus rosmarus divergens* teeth can be used to estimate weaning age. Teeth from 107 walruses were analysed using laser ablation inductively coupled plasma mass spectrometry, and calcium‐normalized ^88^Sr and ^137^Ba concentrations were quantified.
3. For most walruses, both Sr and Ba concentrations exhibited rapid changes in early life. Ba concentrations matched closely with expected patterns in the published literature, rapidly declining from high to low concentrations (typically from ~10 ppm to ~5 ppm). In contrast, Sr exhibited a pattern opposite to that presented in studies of terrestrial mammals, appearing nearly identical to Ba (typically declining from ~400 ppm to ~200 ppm). To explain these findings, we present conceptual models of the factors generating weaning signals in Sr and Ba for terrestrial mammals, as well as a new, hypothetical model for walruses. Both a visual and mathematical approach to weaning age estimation indicated a median weaning age of walruses at the end of the second year of life (in the second dark layer of the tooth cementum), with many walruses estimated to have weaned in their third year of life, and a smaller group weaning in their fourth or fifth year. This is later than expected, given a published estimate of walrus weaning at 18–24 months.
4. These results do not conclusively support the use of tooth Sr and Ba for estimating weaning age in walruses, and further research is warranted to better understand the drivers of the observed patterns of Ba and Sr accumulation in walrus teeth.

## 1 INTRODUCTION

The age at which mammals wean their offspring is indicative of the amount of energy invested in that offspring by the mother (Lee, Majluf, & Gordon, 1991; Trivers, 1972). Milk production is energetically costly, and typically accounts for the greatest proportion of total reproductive energy expenditure (Hanwell & Peaker, 1977; Lee et al., 1991). Because a mother's ability to produce milk is affected by her body condition and ability to acquire nutrients before and during lactation (Loudon, Darroch, & Milne, 1984), weaning age can provide important information about the health and nutritional status of the mother, as well as the availability of resources in the environment (Lee et al., 1991; McMahon, Harcourt, Burton, Daniel, & Hindell, 2017; Trivers, 1972). Finally, factors such as the quantity and/or quality of milk produced by the mother can be used to track differences in reproductive investment between offspring of different sexes, giving insight into reproductive strategies employed by mammals (Hinde, 2009).

Weaning age is difficult to study in wild mammal populations. Though direct observation of age‐at‐weaning is possible for some species, the life histories of many mammals make such observations unfeasible. Similarly, because direct observation is not possible for animals that are no longer living, studying weaning is especially challenging for mammals from historic populations, as well as archaeological and palaeontological assemblages. To address this issue, researchers have developed ways to estimate nursing and weaning histories based on concentrations of trace elements in mammalian teeth (for a brief review, see Tsutaya & Yoneda, 2015). This approach relies on differences in the calcium‐normalized concentrations and bioavailability of strontium (Sr) and barium (Ba) between mother's milk and adult diet (Comar, Wasserman, Ullberg, & Andrews, 1957; Humphrey, Dirks, Dean, & Jeffries, 2008; Sillen & Kavanagh, 1982). By comparing changes in the concentrations of these elements to growth layer groups (GLGs) in the teeth, which accrete annually and preserve trace element concentrations from their time of formation (Fay, 1982; Laws, 1952; Outridge, Veinott, & Evans, 1995), researchers are able to estimate age‐at‐weaning. To date, this method has been used exclusively for primates, including hominids (Austin et al., 2013; Humphrey, 2014; Humphrey, Dean, Jeffries, & Penn, 2008; Humphrey, Dirks, et al., 2008; Smith, Austin, Hinde, Vogel, & Arora, 2017; Tsutaya & Yoneda, 2015), and though Sr and Ba concentrations in homogenized bone and tooth samples have been used as coarse indicators of weaning age in other groups of terrestrial mammals (e.g. Kierdorf, Stoffels, & Kierdorf, 2014; Metcalfe, Longstaffe, & Zazula, 2010), neither approach has been tested for use on marine mammals.

Traditional methods for assessing weaning age are often not appropriate for marine mammals. Observational estimates of nursing duration and age‐at‐weaning are relatively simple to obtain for some pinnipeds (e.g. Steller sea lions, *Eumetopias jubatus*; Maniscalco, 2014). For species that breed in inaccessible habitats, nurse aquatically or are difficult to observe during the lactation period, however, this approach is often unfeasible. Pacific walruses *Odobenus rosmarus divergens* occupy remote areas of the Arctic and sub‐Arctic, and undertake long migratory movements during their extended nursing period (Fay, 1982). Coupled with largely aquatic nursing behaviour, these factors serve to make observational assessment of age‐at‐weaning next to impossible for free ranging walruses. The current understanding of walrus nursing and weaning patterns is based on analysis of stomach contents of animals collected during Alaska Native subsistence harvests (Fay, 1982). This approach provides a rough estimate of the average weaning age for the Pacific walrus population (18–24 months), but, because weaning is a process and stomach contents represent only a snapshot in time, it is subject to a great deal of uncertainty and cannot estimate age‐at‐weaning for individual animals. Stable isotope analysis of tooth GLGs has been used to assess weaning age for a number of marine mammal species (e.g. Knoff, Hohn, & Macko, 2008; Matthews & Ferguson, 2015; Newsome, Etnier, Monson, & Fogel, 2009; York, Thomason, Sinclair, & Hobson, 2008); however, such analyses have not been published for walruses.

Understanding the biology and ecology of Pacific walruses is increasingly important, in light of the rapid changes occurring to their habitat, and the role of this species as a food source for Russian and Alaska Native communities (Jay, Marcot, & Douglas, 2011). Though the decision was recently made not to list the Pacific walrus as threatened or endangered under the U.S. Endangered Species Act (MacCracken, Beatty, Garlich‐Miller, Kissling, & Snyder, 2017), many concerns remain about the impacts that continued sea ice loss and changing Arctic food webs will have on walruses. The ability to estimate weaning age by analysing trace element concentrations in teeth, which are collected during subsistence harvests to monitor the age‐structure of harvested animals and are abundant in museum collections, could help wildlife managers and co‐management groups to maintain a healthy walrus population in the future.

The primary objective of this study was to assess whether calcium‐normalized Sr and Ba concentrations in Pacific walrus tooth cementum can be used to estimate age‐at‐weaning for this species. To accomplish this, elemental concentration data were first examined qualitatively, and compared with expected patterns of accumulation published for other species. Second, weaning age estimates were generated from each walrus’ Sr and Ba data using both a visual (qualitative) and mathematical (quantitative) approach. The presumed weaning ages based on the Sr and Ba for each individual were compared, as were the results of the visual and mathematical estimation methods, to examine the consistency of the age‐at‐weaning results for each walrus. Finally, the presumed ages‐at‐weaning produced as part of this study were compared with values from the published literature.

## 2 MATERIALS AND METHODS

### 2.1 Trace element analysis and data processing

Walrus teeth used for this study (female: *n* = 84, male: *n* = 23) were on loan from the University of Alaska Museum in Fairbanks, Alaska, the National Museum of Natural History, Smithsonian Institution, in Washington DC, and the Alaska Department of Fish & Game. These specimens each represented an individual animal, and were collected at various locations throughout the Pacific walrus range in the Bering and Chukchi seas. Most teeth originated from Alaska Native subsistence harvests, though a small number came from research expeditions. Dates of collection ranged from 1932 to 2016. In preparation for analysis, teeth were sectioned longitudinally using a slow‐speed, water‐cooled saw equipped with a diamond blade, creating a cross section of the centre of the tooth with a thickness of ~1.5 mm. This cross section was then polished using a rotary polishing wheel with a 3,000 grit smoothing disc, rinsed with ultrapure water, and allowed to dry. Specimens were rinsed and dried again immediately prior to trace element analysis.

Trace element analyses were conducted in the Advanced Instrumentation Laboratory at the University of Alaska Fairbanks (UAF), Fairbanks, Alaska. Concentrations of ^88^Sr and ^137^Ba were measured using an Agilent 7500ce Inductively Coupled Plasma Mass Spectrometer (ICP‐MS; fitted with an Agilent 7500cs lens stack to improve sensitivity) coupled with a New Wave UP213 laser. Instrumental precision for the ICP‐MS is reported at ±5%. ^43^Ca was used as an internal standard for these analyses, and all results are reported in parts per million (ppm). Measured element concentrations were compared to a United States Geological Survey microanalytical phosphate standard (MAPS‐4), as well as a National Institute of Standards and Technology Standard Reference Material (SRM 610). Accuracy and precision were estimated by comparing element concentrations measured during ablation of the reference materials (*n* = 363) with reported concentrations. Sr and Ba measurements were both accurate to within 1% of reported values, with a precision (±1 *SD*) of ±4% and ±5%, respectively. Laser transects were ablated at a beam width of 25 μm, at 55% power, with a pulse frequency of 10 Hz, and a transect speed of 5 μm/s. Dwell times were 0.02 s for ^43^Ca, 0.01 s for ^88^Sr and 0.15 s for ^137^Ba. Ablation was conducted at locations that maximized distance from the root of the tooth, where cementum GLGs converge and become distorted, while avoiding areas of tooth wear near the crown, where not all cementum layers are present for sampling. Each transect was ablated from the cementum‐dentin interface (first year of life) to the outer edge of the tooth (final year of life), thereby measuring lifetime changes in element concentrations for each walrus (Fay, 1982).

Trace element data were extracted and processed in Igor Pro version 6.37 using the Iolite software package version 3.0. Statistical analyses were conducted using R version 3.6.3 (R Core Team, 2020) with RStudio version 1.2.5033 (RStudio Team, 2015). Limits of detection were calculated for each analytical run using the standard method applied by Iolite (Longerich, Jackson, & Günther, 1996). Typical limits of detection were 0.18 ppm for Sr and 0.34 ppm for Ba. Element concentrations falling below the limit of detection were replaced with a value of one half the limit of detection (U.S. Environmental Protection Agency, 2000). Data points that were more than 4 *SD* above or below the mean were considered outliers (Tukey, 1977). These were typically single data points believed to represent measurement errors generated during data collection and processing, rather than changes in element concentrations within the tooth, thus they were removed from subsequent analyses.

All specimens used in this study were obtained from museum collections and/or Alaska Native subsistence harvests, thus this work is Institutional Animal Care and Use Committee (IACUC) exempt. Specimens from subsistence harvests were transferred to UAF for analysis under a Letter of Authorization from the United States Fish and Wildlife Service (USFWS) to Dr. L. Horstmann.

### 2.2 Weaning age assignment

After analysis on the ICP‐MS, teeth were photographed under a Leica M165 C optical microscope coupled with a Leica DFC295 camera using reflected light. Level of magnification used when taking photographs varied depending on the size of the tooth, and was selected to maximize visibility of the GLGs. The first five GLGs in the tooth cementum were counted (Fay, 1982; Garlich‐Miller, Stewart, Stewart, & Hiltz, 1993) and their positions marked on the images to denote the positions of the first five years of life on the laser ablation transect. Growth layer groups consist of paired bands of cementum, which accrete onto the tooth annually (Fay, 1982). The terminology used to describe GLGs has not been standardized and differences in the appearance of growth layers under reflected and transmitted light may cause confusion. In this study, the term ‘light layer’ refers to the opaque, hypercalcified layer that accretes during periods of faster growth. This layer represents the period from approximately mid‐April to mid‐December (Clark, Horstmann, & Misarti, 2020b). Under reflected light, this layer appears white, but it is dark under transmitted light. The term ‘dark layer’ refers to the more translucent, hypocalcified layer that is built during periods of slower growth. The dark layer represents the period from around mid‐December to mid‐April (Clark et al., 2020b). This layer appears dark under reflected light, because it allows light to pass through, thus it appears white when using transmitted light. Individually, each light or dark layer is referred to as a growth layer, whereas a pair of light and dark layers, representing 1 year of growth, is referred to as a growth layer group (Laws, 1952). Growth layers were identified and marked collaboratively by three observers (C.T.C., L.H. and N.M.), and the positions of these growth layers were revisited on at least two additional days to confirm their positions on the laser ablation transect. Estimates of overall age (age at death) were also generated for each animal using the methods described in Clark et al. (2020b), and median age estimates were used to calculate approximate birth year for all animals examined in this study (Table S1).

**FIGURE 1:**
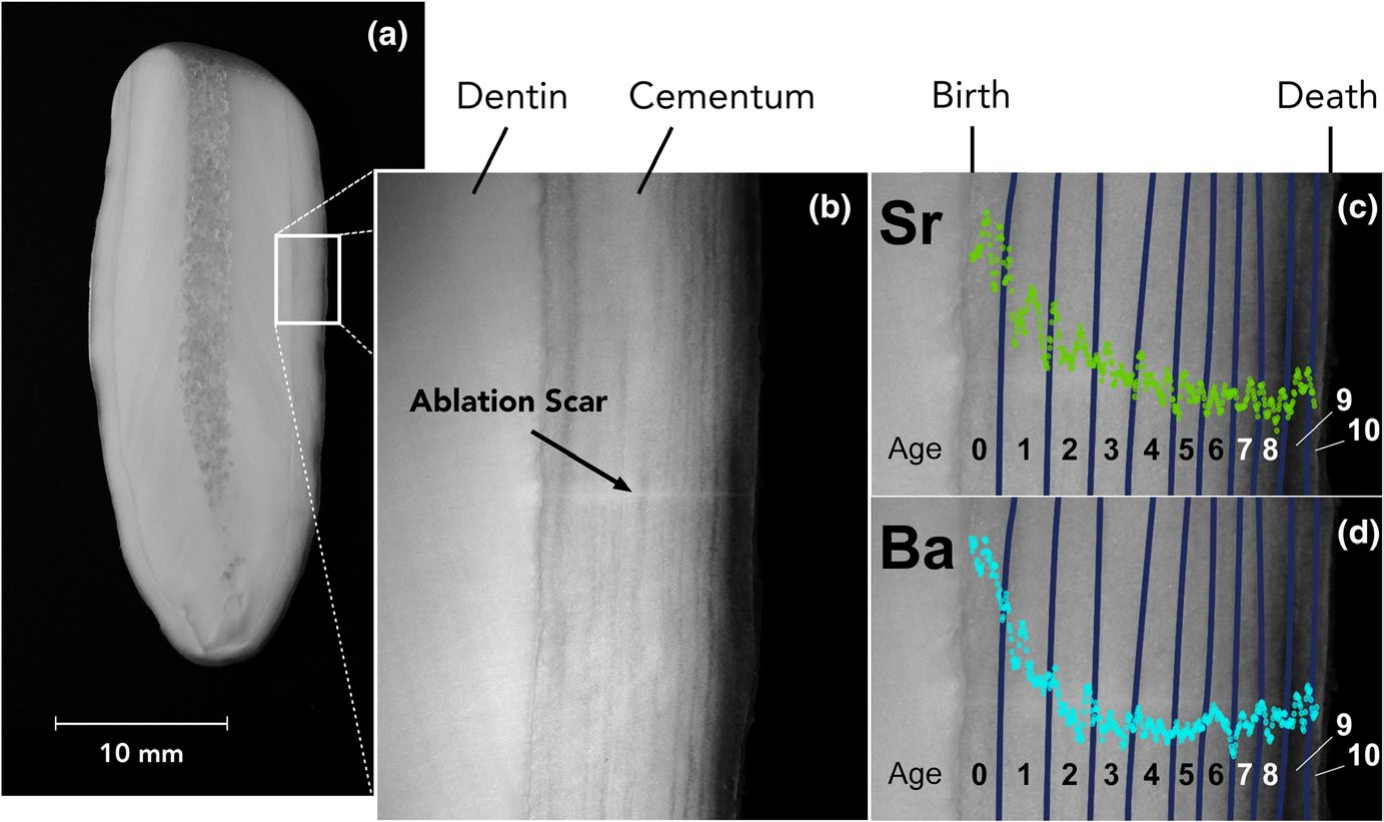
(a) Image of a cross‐sectioned walrus tooth. (b) Image of the site of laser ablation, with annotations marking the dentin, cementum and ablation scar. (c & d) Image of the site of laser ablation, with the dark cementum layers highlighted in dark blue, and the animal's age in years denoted on each growth layer group. Strontium (Sr) and barium (Ba) concentrations (ppm) are overlaid on the images to show how the data align with the growth layers

Analysis of Sr and Ba data for age‐at‐weaning estimation was restricted to the first five years of life. Prior to analysis, data were smoothed using a Savitzky–Golay filter from the r package prospectr (Stevens & Ramirez‐Lopez, 2014) with a window of 15 data points. This approach was chosen because it reduces noise in the data, allowing underlying patterns to be seen more clearly, but results in very little data loss and uses a centred method, thus does not shift the data left or right like some other smoothing methods. Weaning is a process, rather than a single event, beginning with the first intake of non‐milk food items and ending when consumption of mother's milk has ceased completely. For the purposes of this study, a walrus was considered to have weaned at the point where the early life signal in its cementum Sr or Ba time series, associated with consumption of milk, transitioned to relatively stable values that persisted throughout the animal's adult life. The weaning age estimates generated here thus represent the end of the weaning process and the transition to an entirely non‐milk diet. Data were first analysed visually, with a single observer (C.T.C.) identifying patterns in Sr and Ba indicative of weaning (e.g. abrupt decreases/increases in concentration, changes in slope, etc.) based on expected patterns from the published literature (Austin et al., 2013; Humphrey, 2014; Humphrey, Dean, et al., 2008; Humphrey, Dirks, et al., 2008; Smith et al., 2017; Tsutaya & Yoneda, 2015). Because Pacific walrus tooth cementum typically begins growing in the second or third month after birth (Fay, 1982), a signal associated with the onset of nursing (low and stable Sr concentrations/rapidly increasing Ba concentrations) was not expected. Once a suspected weaning signal was selected, a plot of the data was overlaid on an image of the individual tooth from which they were collected, with the *x*‐axis aligned to the laser ablation scar (Figure 1). In this way, the location of the estimated weaning signal could be directly assigned to the growth layer in which it occurred. After visual analysis, weaning estimates were made using a mathematical approach, in which the data were subjected to an iterative method of change point detection using the r package segmented (Muggeo, 2008). This tool detects underlying changes in slope within a time series and, for the purposes of this analysis, was restricted to assigning a single change point within the first five years of an animal's life. Because the mathematical approach was susceptible to both positive and negative changes in slope, and was restricted to assigning only a single change point to the elemental time series, periods of increasing or stable element concentrations at the beginning of an animal's life (relatively rare, and possibly associated with the onset of and/or a period of sustained nursing) were omitted from the Sr and Ba data when applying the mathematical weaning estimation approach. While these variations in the patterns of element accumulation in the first year of life may contain important information, they were excluded for their tendency to interfere with the ability of the segmented regressions to assign a change point associated with the end of weaning and the attainment of an entirely non‐milk diet.

Weaning age data were analysed qualitatively, by comparing estimates of age‐at‐weaning generated by the visual and mathematical estimation methods for Sr and Ba to walrus weaning age estimates from the published literature. The performance of these two different estimation methods was compared by examining the average differences in the predictions generated by the two approaches. Chi‐squared tests were used to examine differences in the weaning age predictions generated for female and male walruses. Significance was assessed using an alpha of 0.05. Weaning age estimates were plotted by approximate year of birth for each individual, and visually examined to determine whether any patterns in estimated age‐at‐weaning existed across the ~100‐year span represented by the walrus teeth in this study. Regional differences in weaning age estimates were not assessed, as only location of harvest/collection was available for the animals in this study. The Pacific walrus population is large and panmictic, with little evidence for internal structure (Beatty et al., 2020). Individual walruses move widely over the species' range (Beatty et al., 2016; Jay, Fischbach, & Kochnev, 2012; Jay, Udevitz, Kwok, Fischbach, & Douglas, 2010), thus location of collection is unlikely to provide useful information about the regions in which an individual walrus spent its life or to reflect association with a distinct group within the larger population.

## 3 RESULTS

**FIGURE 2:**
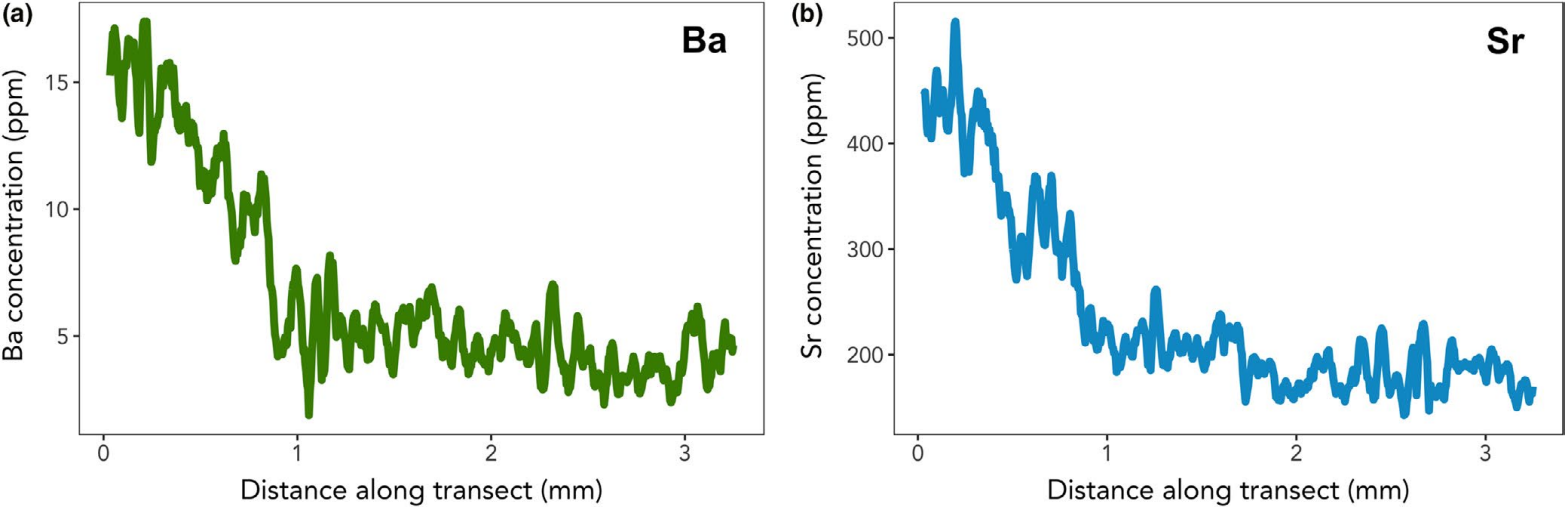
General patterns of accumulation of Ba (panel a, green line) and Sr (panel b, blue line) in Pacific walrus tooth cementum. Both elements exhibited high concentrations in early life, typically declining to low and stable values in adulthood. Each panel represents data from a single individual chosen as a broadly representative example of patterns of element accumulation exhibited by walruses examined for this study

Concentrations of both Sr and Ba in the tooth cementum were above detection limits across the first five GLGs for all walruses examined in this study. Patterns of Ba accumulation in the walrus teeth closely matched those in the published literature (Austin et al., 2013; Smith et al., 2017; Tsutaya & Yoneda, 2015), with animals typically exhibiting high concentrations in early life (typically ~10 ppm), declining to a low and stable (~5 ppm, slope close to zero) values in the animal's later years (Figure 2a). For Sr, the patterns of accumulation observed in the walruses were distinctly different from previously published data for terrestrial animals. Patterns of Sr accumulation in walrus teeth analysed during this study looked nearly identical to those of Ba, with high values (~400 ppm) generally occurring in early life and declining to low values that remained relatively stable (~200 ppm, slope close to zero) throughout adult life (Figure 2b). In light of this unexpected result, all weaning age estimates based on Sr were made using the same criteria expected of the weaning signal in Ba: steep declines in concentration from high values early in life (often decreasing by half in 2–3 years), followed by a distinct change in slope either to a continued, shallower decline or low and stable values (i.e. slope close to zero).

**FIGURE 3:**
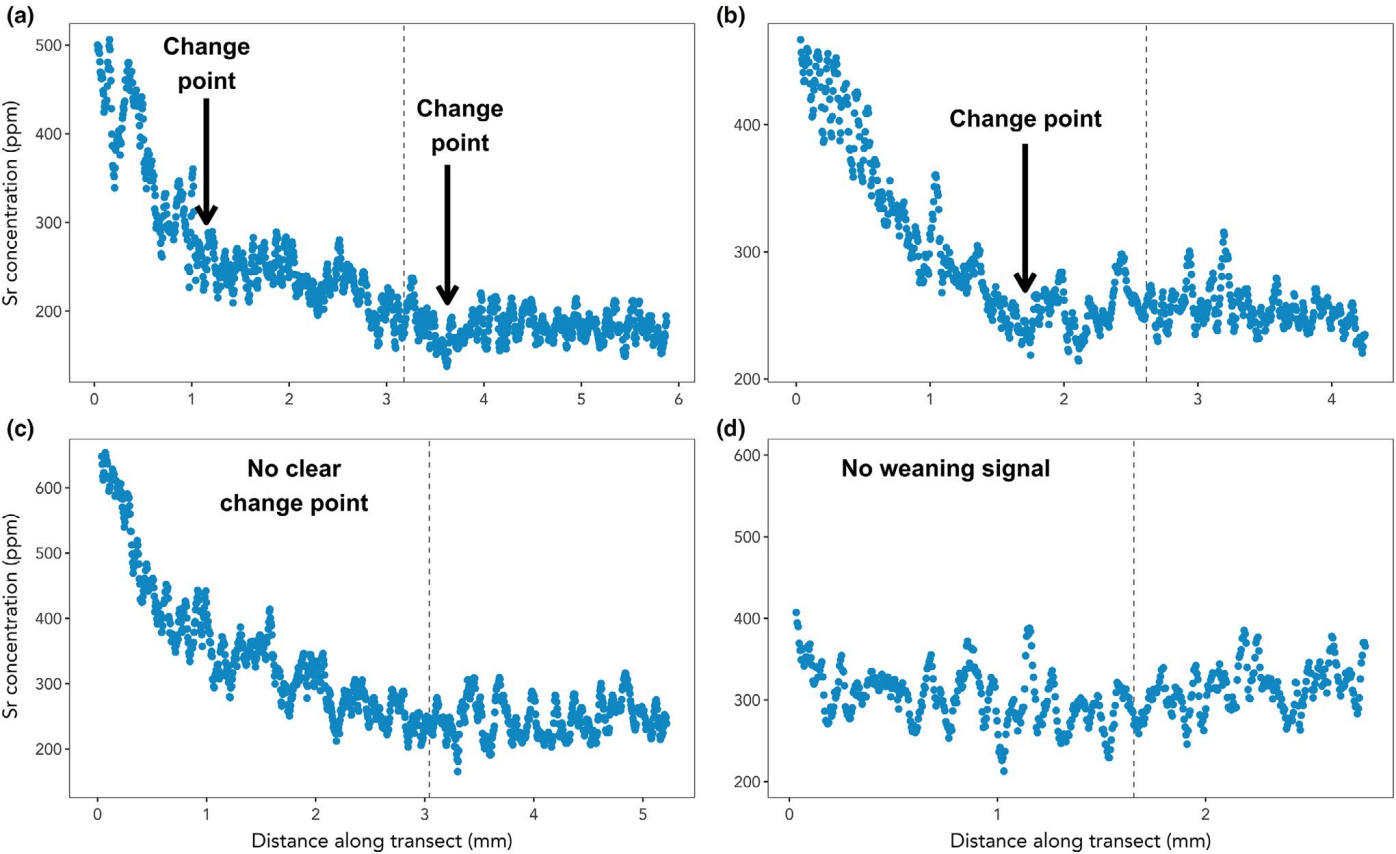
The four typical patterns of Sr and Ba accumulation observed in walrus teeth. Only Sr is included in this figure for brevity, but identical patterns were observed for both elements. (a) Steeply declining elemental concentrations in early life to a change point, after which concentrations declined more slowly, before reaching a second change point and remaining low and stable. (b) Steeply declining elemental concentrations in early life until a single change point is reached, followed by low and stable values. (c) A gentle linear or curvilinear decline to low and stable values, with no clear change point. (d) No consistent decline in Sr or Ba in early life. Vertical dashed lines denote the end of the fifth year of life (cementum layer D5). Data in each panel represent a single individual, chosen to be representative of the four general patterns observed in walrus teeth. Change points in this figure were assigned visually

Though the specific patterns of accumulation of Sr and Ba differed among individual walruses, they could typically be separated into four general groups. In the first group (Sr: 44 animals or 41%; Ba: 33 animals or 32%), the elemental concentrations declined steeply in early life before reaching a change point, after which the decline continued with a shallower slope. The elemental concentrations then continued this slower decline to a second change point, where the decline stopped and the values remained low and stable for the duration of the animal's life (Figure 3a). In the second group (Sr: 46 animals or 43%; Ba: 50 animals or 47%), the concentrations declined sharply in early life, then reached a change point after which they remained consistently low (Figure 3b). In the third group (Sr: 11 animals or 10%; Ba: 6 animals or 6%), no steep decline was present in early life. Instead, elemental concentrations exhibited a linear or curvilinear decline to consistently low values later in life (Figure 3c). Finally, for some animals (Sr: 6 animals or 6%; Ba: 17 animals or 16%), Sr and/or Ba concentrations did not decline in early life and the patterns typically associated with weaning were not present (Figure 3d). The Sr and Ba patterns exhibited by individual walruses were classified into the same group 56% of the time (60 individuals). Of the remaining 47 individuals, 27 (25% of all walruses) exhibited pattern 1 for one element (Sr or Ba) and pattern 2 for the other, whereas the remaining 20 animals (19% of all animals) had some other combination of patterns. Overall, the visual and mathematical estimation methods were able to identify a presumed weaning age more frequently when using Sr to generate the estimates (100 animals or 93%) than when Ba was used (89 animals or 83%).

**FIGURE 4:**
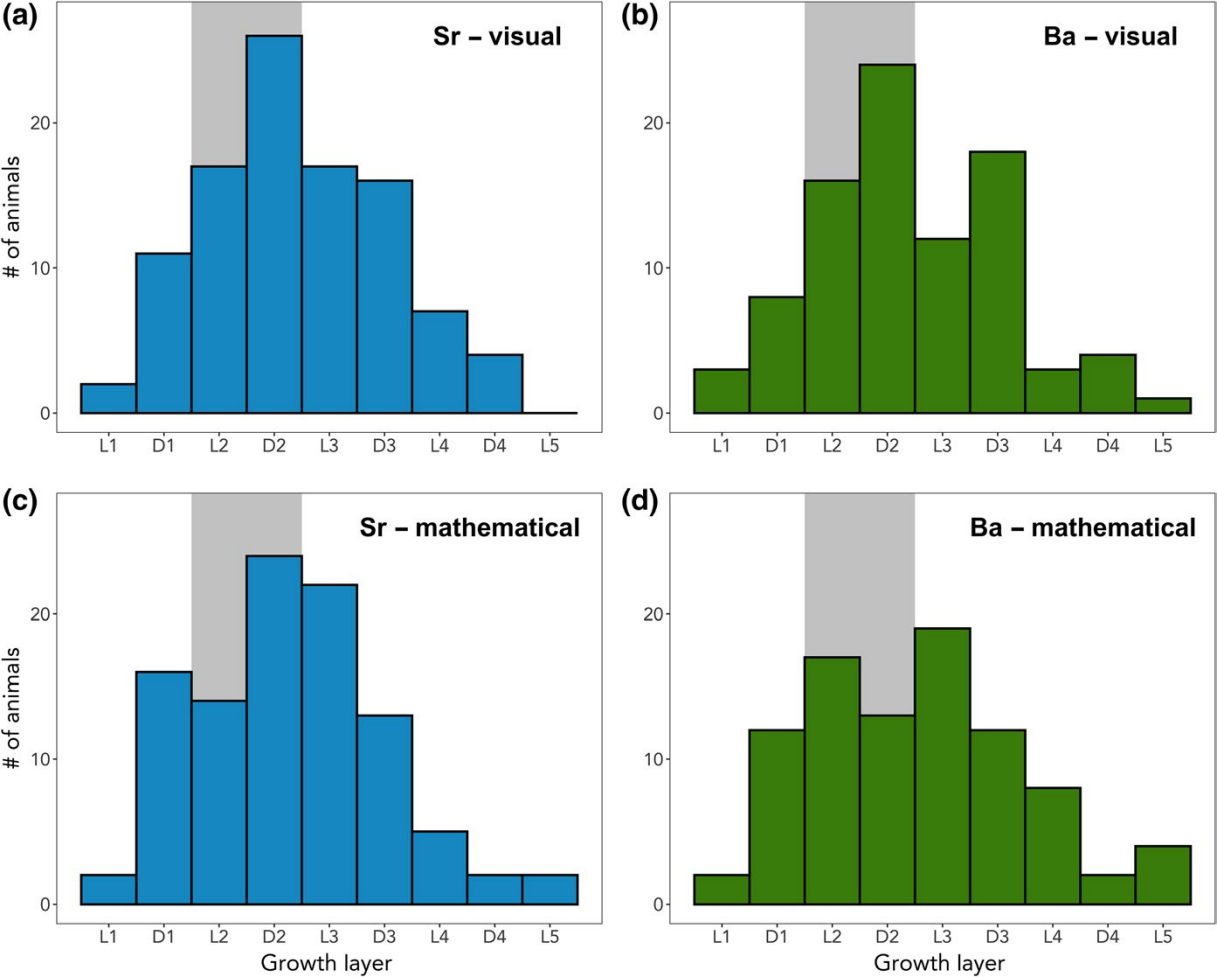
Histograms of Pacific walrus weaning estimates based on Sr (panels a and c) and Ba (panels b and d) concentrations in tooth cementum. Results are shown for visual (panels a and b) and mathematical (panels c and d) weaning age estimation methods. Values on the *x*‐axis represent cementum growth layer groups, with L1 representing the first light layer, D1 representing the first dark layer, and so on until the latest weaning assignments in the fifth light layer (L5). The grey shaded area represents the estimated age‐at‐weaning of 18–24 months from the published literature (Fay, 1982)

When considering only the animals for which presumed weaning age could be estimated (Sr: *n* = 100, Ba: *n* = 89), the visual and mathematical methods provided largely similar results (Table S1). For Sr, the median estimate for age‐at‐weaning was Age 1, in the second dark growth layer (i.e. the second winter of life) for both the visual and mathematical estimates. Overall, the visual (mathematical) age‐at‐weaning estimates based on Sr indicated that 56% (56%) of animals had presumably weaned by the end of their second year of life (Age 1, Dark Layer 2), 89% (91%) by the end of their third year of life (Age 2, Dark Layer 3) and 100% (98%) by the end of their fourth year of life (Age 3, Dark Layer 4; Figure 4a,b). The mathematical approach indicated that a single individual weaned in its fifth year of life (Age 4). Barium‐based estimates of weaning age provided generally similar results. For Ba, the median age‐at‐weaning estimate was also Age 1, in the second dark growth layer, for both methods of estimation. The visual (mathematical) age‐at‐weaning estimates based on Ba indicated that 56% (48%) of walruses had presumably weaned by the end of their second year of life, 90% (83%) by the end of their third year of life and 98% (94%) had weaned by the end of their fourth year of life (Figure 4c,d). The visual approach based on Ba concentrations estimated age‐at‐weaning in the fifth year of life for one animal, whereas the mathematical approach estimated that four walruses weaned in their fifth year.

The estimates of weaning age produced by the visual and mathematical approaches were typically in close agreement. For Sr, the two estimation methods produced identical estimates 71% of the time, results that were within one growth layer (i.e. ~6 months) of one another 90% of the time and within two growth layers (i.e. ~1 year) 94% of the time. The maximum difference between the two estimates was seven growth layers (~3.5 years). For Ba, the visual and mathematical methods produced identical results 55% of the time, results within one growth layer 82% of the time and within two growth layers 89% of the time. The maximum difference between the visual and mathematical estimates for Ba was six growth layers (~3 years). Weaning estimates did not differ between sexes for Sr (Visual: *χ* ^2^(7, *N* = 100) = 10.58, *p* = 0.16; Mathematical: *χ* ^2^(8, *N* = 100) = 10.28, *p* = 0.25) or Ba (Visual: *χ* ^2^(8, *N* = 89) = 6.70, *p* = 0.57; Mathematical: *χ* ^2^(8, *N* = 89) = 11.14, *p* = 0.19), using either the visual or mathematical estimation methods. Visual examination of weaning age estimates plotted by approximate birth year for each individual walrus did not reveal any consistent patterns across elements or between the data produced by the visual and mathematical estimation approaches (Figure S1). The four animals estimated to have been born prior to 1925 were all estimated to have weaned relatively young; however, this may be an artefact of small sample size during this period.

## 4 DISCUSSION

### 4.1 Obstacles to interpretation

Mammalian trace element physiology remains poorly understood, and a number of potentially confounding factors should be considered prior to interpreting the results of this study. The Sr and Ba concentrations in walrus teeth likely result from a series of complex, interacting mechanisms, some of which may mask a weaning signal, while others may generate a signal that might be misinterpreted as an indication of weaning. Studies of macaque *Macaca mulatta* and fossil hominin *Australopithecus africanus* teeth indicate that periods of stress (e.g. moderate to severe illness or nutritional stress) impact tooth mineralization, and may result in the remobilization of skeletal materials, both of which can affect concentrations of trace elements (including Sr and Ba) in the teeth (Austin et al., 2016; Joannes‐Boyau et al., 2019). Such periods of stress in early life might thus change the Sr and Ba concentrations in a way that obscures other physiological or dietary signals, and could perhaps explain why the teeth of some walruses did not appear to contain an Sr or Ba weaning signal. Additionally, it remains unknown whether the incorporation of trace elements into the teeth lags behind their uptake from the diet. Analysis of stable isotopes and trace elements in bone suggests that trace element concentrations in this tissue can lag months behind uptake (Herring, Saunders, & Katzenberg, 1998). However, the lag in bone results primarily from the slow remodeling of this tissue, such that only long term, sustained changes in stable isotope values or trace element concentrations are incorporated (Humphrey, 2014). In contrast, tooth cementum is continually growing and, once accreted, does not undergo significant remodelling or metabolic turnover (Carlson, 1990). Thus, elemental concentrations in the tooth cementum growth layers should represent a snapshot of the time at which each growth layer was accreted. The exact mechanisms by which trace elements are incorporated into the tooth remain unknown, and it is possible that other processes could result in a lag between uptake and incorporation into cementum (e.g. routing of elements through various tissues or metabolic pathways prior to incorporation), but there is currently no evidence that this is the case.

In addition to the potential factors related to trace element physiology that can hinder interpretation of the Sr and Ba data, some of the difficulties of working with tooth GLGs could impact weaning age estimates. For example, identifying the exact positions of individual GLGs is inherently uncertain (Medill, Derocher, Stirling, Lunn, & Moses, 2009), and misidentification of the position of the dark and light cementum layers has the potential to introduce error into the estimates of weaning age. This is particularly true as, to preserve the laser ablation scar (thus the ability to align the trace element data to the GLGs), the walrus teeth examined in this study could not be thin‐sectioned and stained, a technique commonly used to increase the visibility of GLGs and minimize tooth ageing errors (Fancy, 1980). Systematic bias can result in consistently inflated or deflated estimates. In this study, however, analysis of cementum GLGs was restricted to the first five years of life. These first growth layers tend to be wider and more clearly defined than the outer layers, which may be compressed and difficult to see (Fay, 1982; Medill et al., 2009), thus analysing only these layers is likely to have minimized any errors associated with identification of the GLGs. Coupled with the uncertainty of identifying the exact locations of estimated weaning signals in the Sr and Ba time series, however, it is possible that errors associated with defining the exact positions of the cementum growth layers impacted the results of this study.

### 4.2 Accuracy of the Sr and Ba weaning age estimates

Despite the factors discussed above that can potentially confound the estimation of weaning age based on tooth Sr and Ba, the results of this study provide substantial support for the validity of this approach. Several methods for assessing the utility of Sr and Ba concentrations in teeth as indicators of weaning were outlined in the introduction, and each of these provided support for the idea that Ba and Sr in walrus teeth can be used to estimate weaning. First, qualitative examinations revealed a clear signal for both elements in the first growth layers of the walrus tooth cementum. Second, both the visual and mathematical estimation approaches were able to identify presumed weaning ages for nearly all animals examined. Further, within an individual walrus, the estimates of weaning age generated from the visual and mathematical approaches for the Sr data tended to agree closely with one another, and with the estimates produced for the Ba data (Figure 4; Table S1). Together, these results support the validity of this approach for identifying age‐at‐weaning in walruses; however, the weaning age estimates generated as part of this study are, on average, later than would be expected based on the published literature (Fay, 1982), raising questions about their accuracy.

The current understanding of walrus weaning age is based almost exclusively on information from one study. Fay (1982) compiled the few existing reports of Pacific walrus weaning ages, then coupled these with stomach content analysis of ~80 young walruses and his own observational data to produce an overall estimate of age‐at‐weaning. The results of Fay (1982) suggest that most walruses wean between 18 and 24 months of age, though some calves may continue to nurse for an additional year (to ~36 months of age) until their mother gives birth to another calf. This latter conclusion was based on two observations Fay (1982) made of calves aged 34 and 35 months nursing from their mothers, without other, younger calves in the vicinity. Though stomach content and observational analyses are not without their biases and limitations, this remains the best and most referenced estimate of weaning age for this species. The weaning age estimates produced in our study are later than expected, based on Fay's results. The Sr and Ba data suggest that only ≤56% of walruses had weaned by 24 months of age (Figure 4), meaning that more than 40% of the walruses in this study were estimated to have weaned in their third year or beyond. Given the uncertainties and relatively small sample sizes associated with the weaning age estimates from Fay (1982), it remains possible that the estimates of age‐at‐weaning in this study are a better representation of the variability exhibited by the walrus population. Without an effective way to validate the Sr and Ba weaning age estimation methods for walruses, and a better understanding of trace element physiology for this species, it is difficult to say whether the results presented here are accurate estimates of weaning age.

Perhaps some small inference about weaning patterns can be drawn from the average walrus ovulation intervals estimated by Fay (1982). Examinations of 160 reproductive tracts indicated that ~63% females had a 2‐year interval between their two most recent ovulations. This matches with other data sources indicating that most walruses exhibit an average calving interval of 2 years (Fay, 1982). Just under 16% of animals had an ovulation interval of 3 years, while only ~3% had an ovulation interval greater than 3 years. The remaining ~18% had an ovulation interval of 1 year, with the exception of one individual that had ovulated twice within the same year. Fay (1982) suggested that a walrus calf will continue to nurse until its mother gives birth to another, more dependent calf. Operating under this assumption, the ovulation intervals exhibited by female walruses can be used to estimate a ‘maximum nursing duration’ for walrus calves (Figure S2). In the most common case, the mother of a walrus calf born in a given year will become pregnant again the following year, thus the mother will exhibit a 2‐year ovulation interval and will give birth to a second calf 2 years after the first. This would mean the first calf had a window of ~24 months to nurse before it was supplanted by its mother's next offspring. A calf born to a mother that proceeded to exhibit a 3‐year ovulation interval would typically have a window of ~36 months in which it could nurse (Figure S2). Finally, a calf born to a mother that exhibited a subsequent ovulation interval of more than 3 years would, in theory, have greater than 36 months before it was forced to stop nursing. Using the ovulation interval estimates from Fay (1982) as a rough guide, it might be expected that ~63% of calves would be born to mothers that would go on to exhibit a 2‐year ovulation interval, thus around the same proportion of calves would be expected to have weaned by the end of their second year of life. Adding in the Fay (1982) estimate of 16% of females animals with a 3‐year ovulation interval would suggest that ~79% of calves should have weaned by the end of their third year of life. The age‐at‐weaning estimates generated in this study matched fairly well with these predictions, with ≤56% of walruses estimated to have weaned by the end of their second year of life, and ≤90% to have weaned by the end of their third year. There are of course many assumptions and obstacles to this interpretation that make such comparisons problematic. For example, not all ovulations result in pregnancies, and it is unclear how closely the proportions of animals with different ovulation intervals reported by Fay (1982) correspond to average calving intervals, nor is there a straightforward way to incorporate the ~18% of animals that exhibited a 1‐year ovulation interval into these estimates. Some walrus calves wean before the end of their second year, thus the use of a ‘maximum nursing window’ may not be appropriate. Similarly, it is possible that older calves may continue to nurse even after their mother gives birth to another offspring, as has been observed in some otariid species (Maniscalco & Parker, 2009). Despite the uncertainty and required assumptions, broadly speaking, the ovulation interval data appear to support the idea that more walruses may wean after the age of 24 months than was originally predicted by Fay (1982).

### 4.3 Conceptual models of the Sr and Ba weaning signals

Before the accuracy of the walrus weaning estimates generated as part of this study can be assessed further, the substantial difference between the patterns of Sr accumulation exhibited by walruses and those published in the literature for terrestrial animals must be addressed. Of the two elements examined here, Sr has been more thoroughly studied, and the mechanisms responsible for generating the Sr weaning signal in mammalian teeth are thought to be fairly well understood, at least for terrestrial mammals. The fact that Sr closely follows the patterns exhibited by Ba, which even in terrestrial mammals is poorly understood, suggests the existence of important differences in trace metal physiology and metabolism between walruses and primates, and possibly between marine and terrestrial mammals.

**FIGURE 5:**
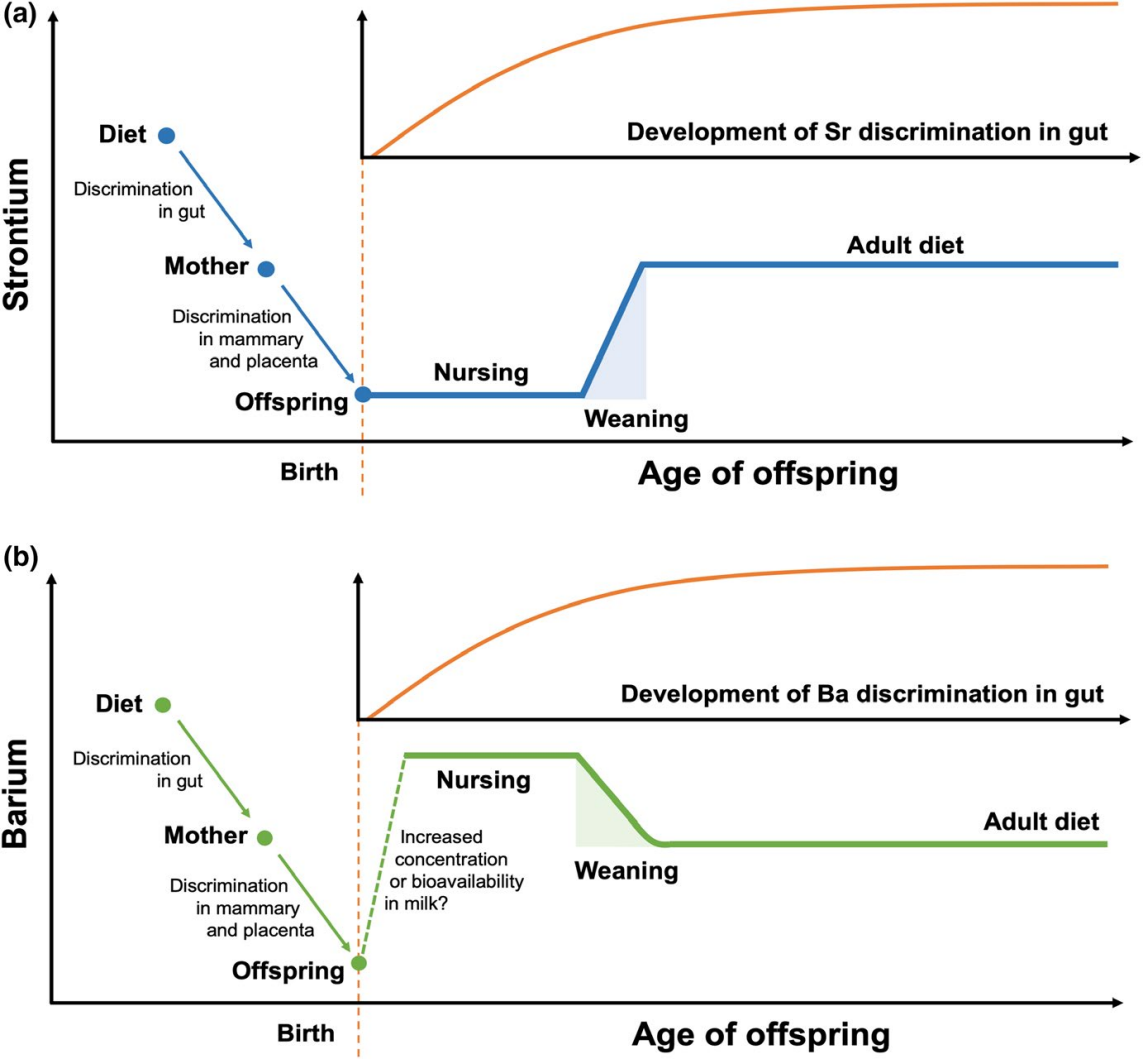
Conceptual models of expected weaning signals in tooth strontium (panel a) and barium (panel b) and their hypothesized drivers, based on data and concepts in the published literature for terrestrial animals (Austin et al., 2013; Humphrey, 2014; Humphrey, Dean, et al., 2008; Humphrey, Dirks, et al., 2008; Smith et al., 2017; Tsutaya & Yoneda, 2015). The inset panel with the orange curve in each diagram represents the expected development of discrimination against strontium or barium during uptake of nutrients from the gut. The vertical, dashed orange line denotes birth. The shaded areas represent the weaning period, from its beginning with the first incorporation of non‐milk foods, to its end, when the offspring consumes an entirely non‐milk diet

For terrestrial mammals, the Sr weaning signal is believed to be generated primarily by differences in the concentration of Sr in mother's milk and that of adult diet (Comar et al., 1957; Sillen & Kavanagh, 1982). Discrimination against strontium (in favour of calcium) occurs in the gut, across the placenta and in the mammary during milk production (Comar et al., 1957; Krachler, Rossipal, & Micetic‐Turk, 1999; Lough, Rivera, & Comar, 1963; Rossipal, 2000). As a result, the calcium‐normalized Sr concentrations in the mother's tissues are expected to be lower than the concentrations found in her diet. The Sr concentrations in the tissues of the foetus should be lower still (Figure 5a). At birth, when the offspring begins nursing, the Sr concentrations in its tissues remain low due to the discrimination against Sr in the mammary. Another important factor in this conceptual model is the development of gut discrimination by the offspring. At birth, discrimination against Sr in the gut of young mammals is limited or nonexistent, and this ability develops slowly with age (Comar et al., 1957; Lough et al., 1963; Peek & Clementz, 2012a). In humans, it develops within the first decade of life (Lough et al., 1963; Sillen & Smith, 1984). Prior to the development of gut discrimination, Sr concentrations in the tissues of young terrestrial mammals should thus be similar to the concentrations found in their diet (mother's milk, for nursing animals), and the development of gut discrimination plays an important role in shaping the pattern of Sr accumulation as the young mammal weans and eventually shifts to a non‐milk diet.

The mechanisms driving the Ba weaning signal in terrestrial mammals are poorly understood. Like Sr, this element experiences discrimination during uptake from the gut (Schroeder, Tipton, & Nason, 1972). Additionally, there are indications that this element experiences discrimination across the placenta and in the mammary during the formation of milk (Austin et al., 2013; Krachler et al., 1999; Peek & Clementz, 2012a). At birth, a young terrestrial mammal is thus expected to have low Ba concentrations in its tissues, and might follow a pattern similar to that described in the literature for Sr; however, studies examining Ba concentrations in young terrestrial mammals have found high concentrations during breastfeeding (e.g. Austin et al., 2013; Smith et al., 2017), which decline to a low and stable value as the animal weans (Figure 5b). In humans, umbilical cord blood has low Ba concentrations, whereas those of exclusively breastfed infants are high, and serum Ba values from adults are intermediate (Krachler et al., 1999). The increased Ba concentrations in young terrestrial mammals may be related to the bioavailability of Ba in the milk, as opposed to the concentration of Ba it contains (Smith et al., 2017; Tsutaya & Yoneda, 2015). Casein digestion releases phosphopeptides, which increase the absorption efficiency of divalent cations like Ca, Sr and Ba (Bouhallab & Bouglé, 2004; Jovaní, Barberá, & Farré, 2001). In contrast to milk, the majority of the Ba found in plant and animal tissues is thought to have low bioavailability (Austin et al., 2013), thus Ba from food items in the adult diet may not be readily incorporated into a consumer's tissues. The relatively high bioavailability in milk could help explain the patterns of Ba accumulation observed in the walrus teeth examined in this study; however, this hypothesis raises further questions about why the Sr and Ba weaning signals exhibit opposite patterns in terrestrial mammals, given that the same phenomenon thought to increase the bioavailability of Ba in milk should also increase Sr bioavailability. It is possible that this difference can be explained by more extreme discrimination against Sr in the mother's body (though Ba may actually experience stronger discrimination than Sr, see Burton, Price, & Middleton, 1999), or by a greater increase in bioavailability in the milk for Ba than for Sr.

**FIGURE 6:**
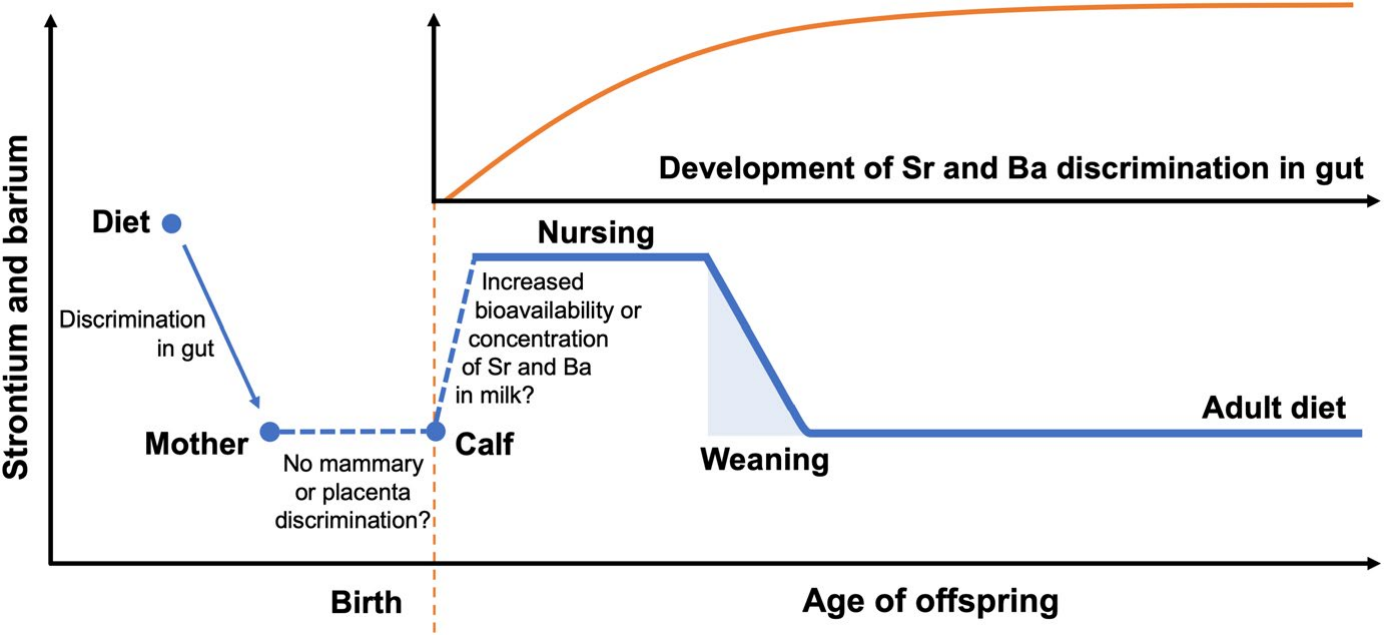
Conceptual models of expected weaning signals in Pacific walrus tooth strontium and barium and their hypothesized drivers, based on the results of this study, as well as data and concepts in the published literature (Austin et al., 2013; Humphrey, 2014; Humphrey, Dean, et al., 2008; Humphrey, Dirks, et al., 2008; Smith et al., 2017; Tsutaya & Yoneda, 2015). The inset panel with the orange curve in each diagram represents the expected development of discrimination against strontium or barium during uptake of nutrients from the gut. The vertical, dashed orange line denotes birth. The shaded area represents the weaning period, from its beginning with the first incorporation of non‐milk foods, to its end, when the offspring consumes an entirely non‐milk diet

For the walruses in this study, it appears that similar mechanisms are driving the patterns observed in Sr and Ba. The elevation of the concentrations of both elements in early life means that walrus calves (a) received greater concentrations of these elements than were found in their mothers' bodies, (b) Sr and Ba bioavailability in milk was greater than that of their adult diets or (c) some combination of these two factors. There is a dearth of research describing trace element transfer from marine mammal mothers to their offspring; however, a study of grey seals *Halichoerus grypus* examined trace metal transfer from mothers to pups and concluded that discrimination against non‐essential trace metals at the placenta and mammary is weak or absent for this species (Habran, Pomeroy, Debier, & Das, 2013). This study did not examine Sr or Ba, so it is unclear whether their findings also apply to these elements, nor is it certain whether their results are transferrable to walruses. If they are, it would mean that, at birth, walrus calves would have similar elemental concentrations to their mothers. Coupled with increased bioavailability in milk, this could cause both Sr and Ba to exhibit patterns of accumulation during nursing and weaning like those observed in the walruses in this study (Figure 6). It is also possible that there is some mechanism concentrating Sr and Ba in walrus milk; however, there is currently no evidence to support this. The difference in baseline Sr concentrations between marine and terrestrial systems may be responsible for the apparent differences in the way walruses and terrestrial mammals transfer this element between mother and young. Marine systems are relatively rich in Sr and, despite discrimination against Sr resulting in ‘biopurification’ of this element with increasing trophic level, marine mammals typically have higher calcium‐normalized Sr concentrations in their tissues than their terrestrial counterparts (Peek & Clementz, 2012b). These elevated baseline Sr concentrations in marine food webs may have resulted in differences in the way marine mammals deal with Sr (uptake, transfer to offspring, excretion, etc.), and could be the reason why the observed patterns of Sr accumulation differ between walruses and terrestrial species; however, much remains to be learned about the role of trace elements in marine mammal physiology and further work is warranted.

Closer examination of the specific patterns of accumulation of Sr and Ba in walrus teeth may provide further insight into the mechanisms at work. Around 30%–40% of animals in this study exhibited two distinct change points in their tooth Sr or Ba concentrations (Figure 4a). It is possible that the earlier of these change points represents weaning, whereas the change in slope later in life occurred as a result of the attainment of fully mature gut discrimination. A larger group of animals (~40%–50%) exhibited only one change point (Figure 4b), perhaps indicating that weaning and the attainment of mature gut discrimination coincided for these animals. It is also possible that one of the signals was more pronounced and masked the other, making it undetectable. Finally, a small number of walruses (~5%–10%) exhibited only a slow, tapering decline in Sr and/or Ba concentrations. For these animals, the weaning signal may have been nonexistent or completely overridden by the development of gut discrimination. Alternatively, it is possible these animals began foraging on invertebrates very early in life. Fay (1982) reports walrus calves with invertebrates in their stomachs 6–7 months after birth. If these individuals had already reached or were nearing the end of the weaning process and switched to a diet consisting entirely of non‐milk foods, any weaning signal would be expected to appear within the first light layer, and would be difficult to detect.

## 5 CONCLUSIONS

Though more direct validation is required before the accuracy of the results of this study can be properly assessed, the combined evidence presented here indicates that the Sr and Ba weaning age estimation method holds promise as a tool for use in walruses, and perhaps in other marine mammals. These results also highlight important differences in trace element physiology of Pacific walruses and those in the published literature for primates. This type of spatially explicit, high‐resolution analysis of trace element concentrations in animal tissues has already provided information about animal physiology, life history and migratory movements (Alibert & McCulloch, 1997; Clark et al., 2020b; Outridge & Stewart, 1999; Secor, Henderson‐Arzapalo, & Piccoli, 1995; Thompson et al., 2003), and will no doubt continue to prove valuable. Future research pairing trace element measurements with other techniques, such as stable isotope or hormone analyses, may be particularly fruitful. Closer examination of the earliest portion of the Sr and Ba records may provide useful information about the age at which non‐milk foods are introduced into the diet of young walruses. That said, a better understanding of the variability in the timing of tooth growth and the accretion of the first layer of cementum is critical to interpreting changes that occur within the first year of life. Most importantly, further study of the processes responsible for determining trace element concentrations in animal tissues is needed to improve the interpretability of this field of research.

## AUTHORS' CONTRIBUTIONS

C.T.C., L.H. and N.M. conceptualized and designed the study; C.T.C. conducted the laboratory work and data processing; C.T.C., L.H. and N.M. identified and counted tooth growth layer groups; C.T.C. identified weaning age estimates; C.T.C., L.H. and N.M. wrote the manuscript.

### Peer Review

The peer review history for this article is available at https://publons.com/publon/10.1111/2041‐210X.13482.

## Supporting information

Supplementary Material

## Data Availability

All data used for this research are deposited in the Dryad Digital Repository https://doi.org/10.5061/dryad.7h44j0zs4 (Clark, Horstmann, & Misarti, 2020a). Specimens used in this research are archived at the University of Alaska Museum, Fairbanks, Alaska, and the United States National Museum, Smithsonian Institution.
