# Supplementary material for: Evaluating tooth strontium and barium as indicators of weaning age in Pacific walruses
Source: Methods Ecol Evol. 2020 Sep 28;11(12):1626–38. doi: 10.1111/2041-210X.13482 (PMC7756818; doi:10.1111/2041-210X.13482)
Supplement: Supplementary file 1 — Supplementary Material [file MEE3-11-1626-s001.docx]

Table S1. Data for Pacific walrus tooth specimens used in this study, including specimen ID, sex, median tooth age estimate, collection year, and estimated birth year, as well as visual and mathematical weaning age estimates based on strontium (Sr) and barium (Ba). Finally, the overall patterns exhibited by the Sr and Ba time series were classified into four general groups (1–4), and the group assignments for each individual are included in the last two columns. University of Alaska Museum catalog numbers begin with the prefix “UAM:Mamm:”, whereas United States National Museum (USNM) catalog numbers are preceded by “USNM”, and Alaska Department of Fish & Game (ADFG) IDs begin with “S”. Weaning age estimates represent the growth layer in which the animal was estimated to have completed weaning, with growth layers “L1” and “D1” representing the first pair of light and dark growth layers (first year of life), “L2” and “D2 representing the second pair of growth layers (second year of life), and so on. “NA” means an estimate could not be made for that element/estimation method.

| **Catalog**  **Number/ID** | **Sex** | **Median**  **Age Est.** | **Coll. Year** | **Est. Birth Year** | **Sr Vis.**  **Est.** | **Sr Math. Est.** | **Ba Vis. Est.** | **Ba Math. Est.** | **Sr Pat.** | **Ba Pat.** |
| --- | --- | --- | --- | --- | --- | --- | --- | --- | --- | --- |
| UAM:Mamm:12084 | M | 24 | 1978 | 1954 | L4 | L3 | D3 | D3 | 1 | 1 |
| UAM:Mamm:11711 | M | 26 | 1963 | 1937 | L3 | D3 | D2 | L3 | 2 | 2 |
| UAM:Mamm:16588 | F | 15 | 1933 | 1918 | D2 | L2 | L2 | L2 | 1 | 2 |
| UAM:Mamm:11709 | F | 22 | 1970 | 1948 | L3 | L3 | L2 | L3 | 1 | 1 |
| UAM:Mamm:10538 | F | 15 | 1973 | 1958 | L4 | L4 | D3 | L2 | 1 | 1 |
| UAM:Mamm:5012 | F | 13 | 1954 | 1941 | L2 | D1 | L2 | L2 | 1 | 2 |
| UAM:Mamm:12086 | M | 20 | 1979 | 1959 | D3 | D3 | D3 | D3 | 3 | 1 |
| UAM:Mamm:16593 | F | 13 | 1932 | 1919 | NA | NA | NA | NA | 4 | 4 |
| UAM:Mamm:11702 | F | 8 | 1956 | 1948 | L2 | D2 | L2 | D2 | 2 | 2 |
| UAM:Mamm:12082 | M | 21 | 1978 | 1957 | D2 | D2 | L3 | L3 | 3 | 3 |
| UAM:Mamm:3382 | M | 10 | 1932 | 1922 | L1 | L1 | L1 | L1 | 1 | 1 |
| UAM:Mamm:11684 | F | 10 | 1972 | 1962 | L3 | L3 | L3 | L3 | 2 | 2 |
| UAM:Mamm:11698 | F | 10 | 1959 | 1949 | L3 | L3 | L4 | L4 | 2 | 2 |
| UAM:Mamm:11685 | F | 9 | 1972 | 1963 | D2 | D2 | D2 | D3 | 2 | 2 |
| UAM:Mamm:11703 | M | 23 | 1956 | 1933 | L2 | L2 | D3 | L5 | 2 | 2 |
| UAM:Mamm:11704 | F | 13 | 1958 | 1945 | L2 | D2 | L4 | L4 | 2 | 2 |
| UAM:Mamm:11693 | F | 4 | 1966 | 1962 | D2 | L3 | D2 | L3 | 1 | 1 |
| UAM:Mamm:16591 | F | 18.5 | 1932 | 1913.5 | D1 | D1 | D1 | D1 | 1 | 2 |
| UAM:Mamm:11691 | F | 10 | 1957 | 1947 | D3 | L5 | L5 | L5 | 2 | 2 |
| UAM:Mamm:16586 | F | 19 | 1933 | 1914 | L2 | D1 | L2 | L1 | 1 | 1 |
| UAM:Mamm:11699 | M | 5 | 1969 | 1964 | D2 | D2 | D2 | D1 | 1 | 1 |
| UAM:Mamm:12073 | M | 9 | 1978 | 1969 | D2 | D2 | D2 | L4 | 2 | 2 |
| UAM:Mamm:11686 | F | 7.5 | 1962 | 1954.5 | L4 | D2 | L3 | D1 | 3 | 3 |
| UAM:Mamm:12075 | M | 16 | 1978 | 1962 | D2 | D2 | L2 | L2 | 2 | 1 |
| UAM:Mamm:11683 | M | 8 | 1965 | 1957 | D4 | D4 | D1 | D1 | 2 | 1 |
| UAM:Mamm:12071 | M | 17 | 1977 | 1960 | L2 | L2 | NA | NA | 2 | 4 |
| UAM:Mamm:12079 | M | 16 | 1978 | 1962 | L3 | L3 | D3 | L3 | 2 | 2 |
| UAM:Mamm:12081 | M | 17 | 1978 | 1961 | D1 | D2 | NA | NA | 2 | 4 |
| UAM:Mamm:125315 | F | 22 | 2014 | 1992 | D2 | D2 | L2 | L2 | 1 | 1 |
| UAM:Mamm:129168 | M | 15 | 2014 | 1999 | D1 | D1 | D1 | D4 | 3 | 1 |
| UAM:Mamm:125324 | M | 16 | 2014 | 1998 | D2 | D2 | D2 | D2 | 2 | 2 |
| UAM:Mamm:125328 | F | 16 | 2014 | 1998 | D3 | D3 | D3 | D3 | 1 | 1 |
| UAM:Mamm:125329 | M | 21 | 2014 | 1993 | D3 | D3 | NA | NA | 2 | 4 |
| UAM:Mamm:125287 | F | 15 | 2014 | 1999 | D2 | D2 | L2 | L2 | 1 | 2 |
| UAM:Mamm:125289 | F | 17 | 2014 | 1997 | D2 | D2 | D3 | L2 | 1 | 1 |
| UAM:Mamm:125291 | F | 12 | 2014 | 2002 | L2 | D1 | D2 | L2 | 1 | 1 |
| USNM 287994 | M | 21 | 1958 | 1937 | D4 | L1 | D3 | L4 | 3 | 2 |
| USNM 324983 | M | 18 | 1962 | 1944 | D3 | D1 | D3 | D3 | 1 | 2 |
| S15-0013 | M | 14 | 2015 | 2001 | NA | NA | NA | NA | 4 | 4 |
| UAM:Mamm:129414 | F | 15 | 2015 | 2000 | D1 | D1 | L1 | L4 | 3 | 2 |
| UAM:Mamm:99597 | F | 10 | 2005 | 1995 | L3 | L3 | L3 | L3 | 1 | 2 |
| UAM:Mamm:131823 | F | 17 | 2016 | 1999 | D3 | D3 | D1 | D1 | 2 | 2 |
| UAM:Mamm:131819 | F | 18 | 2016 | 1998 | D1 | D1 | NA | NA | 1 | 4 |
| UAM:Mamm:131817 | F | 16 | 2016 | 2000 | L4 | L4 | L2 | D1 | 2 | 1 |
| UAM:Mamm:131824 | F | 15 | 2016 | 2001 | D3 | D3 | D2 | D4 | 2 | 2 |
| UAM:Mamm:131821 | F | 20 | 2016 | 1996 | L2 | L2 | D2 | D2 | 2 | 2 |
| UAM:Mamm:130620 | F | 15.5 | 1987 | 1971.5 | L3 | L3 | D2 | D2 | 1 | 1 |
| UAM:Mamm:130621 | F | 12 | 1987 | 1975 | D2 | L2 | D2 | L2 | 2 | 2 |
| UAM:Mamm:130622 | F | 13.5 | 1987 | 1973.5 | D3 | D3 | D3 | D3 | 2 | 2 |
| UAM:Mamm:130623 | F | 15.5 | 1987 | 1971.5 | L2 | L2 | NA | NA | 2 | 4 |
| UAM:Mamm:130624 | F | 20 | 1987 | 1967 | D3 | D3 | D2 | L3 | 2 | 1 |
| UAM:Mamm:108058 | F | 15 | 2006 | 1991 | L2 | L2 | L2 | L2 | 1 | 1 |
| UAM:Mamm:128075 | F | 13.5 | 2012 | 1998.5 | D2 | D2 | D2 | L3 | 1 | 2 |
| UAM:Mamm:128078 | F | 13.5 | 2012 | 1998.5 | D3 | D3 | D3 | D3 | 2 | 1 |
| UAM:Mamm:128086 | F | 12 | 2012 | 2000 | L2 | L2 | D1 | D1 | 1 | 1 |
| UAM:Mamm:130202 | F | 15 | 1981 | 1966 | D3 | L4 | L3 | D2 | 2 | 2 |
| UAM:Mamm:90729 | F | 19 | 1979 | 1960 | D3 | D3 | L3 | L3 | 2 | 2 |
| UAM:Mamm:108129 | F | 15 | 1994 | 1979 | D2 | L3 | L1 | L1 | 2 | 1 |
| UAM:Mamm:121084 | F | 17 | 1960 | 1943 | L3 | L3 | D2 | D2 | 2 | 2 |
| UAM:Mamm:106394 | F | 13 | 1999 | 1986 | L4 | D2 | D3 | L4 | 3 | 3 |
| UAM:Mamm:127992 | F | 11 | 2008 | 1996 | D1 | D1 | D2 | L3 | 1 | 1 |
| UAM:Mamm:121109 | F | 14 | 1960 | 1946 | L3 | L3 | L3 | D2 | 3 | 3 |
| UAM:Mamm:130362 | M | 16 | 1987 | 1971 | D2 | L3 | D2 | L3 | 1 | 2 |
| UAM:Mamm:44164 | F | 12 | 1975 | 1963 | L2 | L2 | L2 | L2 | 1 | 2 |
| UAM:Mamm:129607 | F | 14 | 1987 | 1973 | L3 | L3 | D2 | L3 | 2 | 2 |
| UAM:Mamm:107472 | F | 12 | 2001 | 1989 | D2 | D2 | L3 | L3 | 1 | 2 |
| UAM:Mamm:108166 | M | 10 | 2006 | 1996 | D1 | D1 | L3 | D2 | 1 | 2 |
| UAM:Mamm:106612 | M | 17 | 1995 | 1978 | L2 | D1 | L2 | L2 | 2 | 2 |
| UAM:Mamm:108259 | F | 22 | 1992 | 1970 | D2 | D2 | D2 | D2 | 2 | 2 |
| UAM:Mamm:43591 | F | 14.5 | 1975 | 1960.5 | D4 | D4 | D4 | D2 | 2 | 1 |
| UAM:Mamm:10639 | M | 15 | 1996 | 1981 | D3 | L3 | D3 | D3 | 1 | 2 |
| UAM:Mamm:107053 | F | 16 | 1999 | 1983 | NA | NA | NA | NA | 4 | 4 |
| UAM:Mamm:121047 | F | 14 | 1960 | 1946 | L2 | D2 | L3 | L3 | 1 | 2 |
| UAM:Mamm:121060 | F | 8 | 1960 | 1952 | D4 | L5 | D4 | L5 | 3 | 3 |
| UAM:Mamm:121145 | F | 14 | 1960 | 1946 | L2 | L2 | L3 | L3 | 1 | 1 |
| UAM:Mamm:121192 | F | 12 | 1960 | 1948 | D1 | D1 | L3 | L3 | 3 | 2 |
| UAM:Mamm:121196 | F | 12 | 1960 | 1948 | L3 | L3 | NA | NA | 1 | 4 |
| UAM:Mamm:121197 | F | 10 | 1960 | 1950 | D2 | D1 | NA | NA | 2 | 4 |
| UAM:Mamm:43554 | F | 11 | 1975 | 1964 | D1 | L2 | D2 | D2 | 1 | 2 |
| UAM:Mamm:43588 | F | 12.5 | 1975 | 1962.5 | L2 | D2 | D1 | D1 | 1 | 1 |
| UAM:Mamm:43907 | F | 21 | 1975 | 1954 | D2 | D2 | D3 | L4 | 1 | 2 |
| UAM:Mamm:44168 | F | 10 | 1975 | 1965 | L4 | L4 | L4 | L4 | 1 | 2 |
| UAM:Mamm:129526 | F | 12 | 1987 | 1975 | D2 | L3 | D2 | D2 | 2 | 2 |
| UAM:Mamm:129985 | F | 20 | 1987 | 1967 | L3 | D3 | L3 | L3 | 2 | 2 |
| UAM:Mamm:106514 | F | 11 | 1999 | 1988 | D3 | D3 | D2 | L3 | 2 | 2 |
| UAM:Mamm:106758 | F | 12 | 1996 | 1984 | D1 | D1 | D1 | L2 | 2 | 2 |
| UAM:Mamm:107955 | F | 12 | 1993 | 1981 | L3 | L3 | D4 | L5 | 1 | 2 |
| UAM:Mamm:108127 | F | 28 | 1995 | 1967 | D2 | D2 | D4 | L3 | 1 | 1 |
| UAM:Mamm:108225 | F | 10 | 1996 | 1986 | D2 | D2 | D1 | D1 | 2 | 1 |
| UAM:Mamm:108227 | F | 4.5 | 2000 | 1995.5 | L3 | L3 | D2 | D1 | 2 | 1 |
| UAM:Mamm:107995 | F | 15 | 2007 | 1992 | L4 | L4 | NA | NA | 2 | 4 |
| UAM:Mamm:107996 | F | 10 | 2007 | 1997 | D3 | D3 | D3 | D3 | 2 | 2 |
| UAM:Mamm:127965 | F | 7 | 2008 | 2001 | D2 | D2 | D2 | L3 | 2 | 2 |
| UAM:Mamm:127975 | F | 10 | 2008 | 1998 | L2 | L2 | L2 | L2 | 1 | 1 |
| UAM:Mamm:107117 | F | 16 | 2002 | 1986 | L2 | D3 | L2 | L2 | 3 | 1 |
| UAM:Mamm:108785 | F | 20 | 2000 | 1980 | D3 | L3 | D3 | D3 | 2 | 1 |
| UAM:Mamm:108964 | F | 18.5 | 2002 | 1983.5 | D1 | D1 | NA | NA | 1 | 4 |
| UAM:Mamm:108984 | F | 21 | 2002 | 1981 | D2 | D2 | NA | NA | 2 | 4 |
| UAM:Mamm:109020 | F | 15 | 2002 | 1987 | L3 | L5 | L2 | L2 | 2 | 2 |
| UAM:Mamm:109028 | F | 15 | 2002 | 1987 | D1 | D1 | L2 | D2 | 1 | 3 |
| UAM:Mamm:109059 | F | 17 | 2002 | 1985 | D3 | D3 | NA | NA | 1 | 4 |
| UAM:Mamm:109077 | F | 13 | 2002 | 1989 | L3 | L3 | D3 | L3 | 1 | 1 |
| UAM:Mamm:109152 | F | 18 | 2002 | 1984 | NA | NA | NA | NA | 4 | 4 |
| UAM:Mamm:109212 | F | 14 | 2002 | 1998 | NA | NA | NA | NA | 4 | 4 |
| UAM:Mamm:109221 | F | 17 | 2002 | 1985 | NA | NA | L2 | L2 | 4 | 2 |
| UAM:Mamm:110003 | F | 19 | 2002 | 1983 | D2 | D2 | NA | NA | 1 | 4 |
| UAM:Mamm:110238 | F | 10 | 2002 | 1992 | D2 | D1 | D2 | D1 | 1 | 1 |

Figure S1. Weaning age estimates for Pacific walruses plotted by approximate birth year. Age-at-weaning was estimated from strontium (Sr; Panel A) and barium (Ba; Panel B) data using a visual approach, as well as a mathematical method (Sr: Panel C; Ba: Panel D).


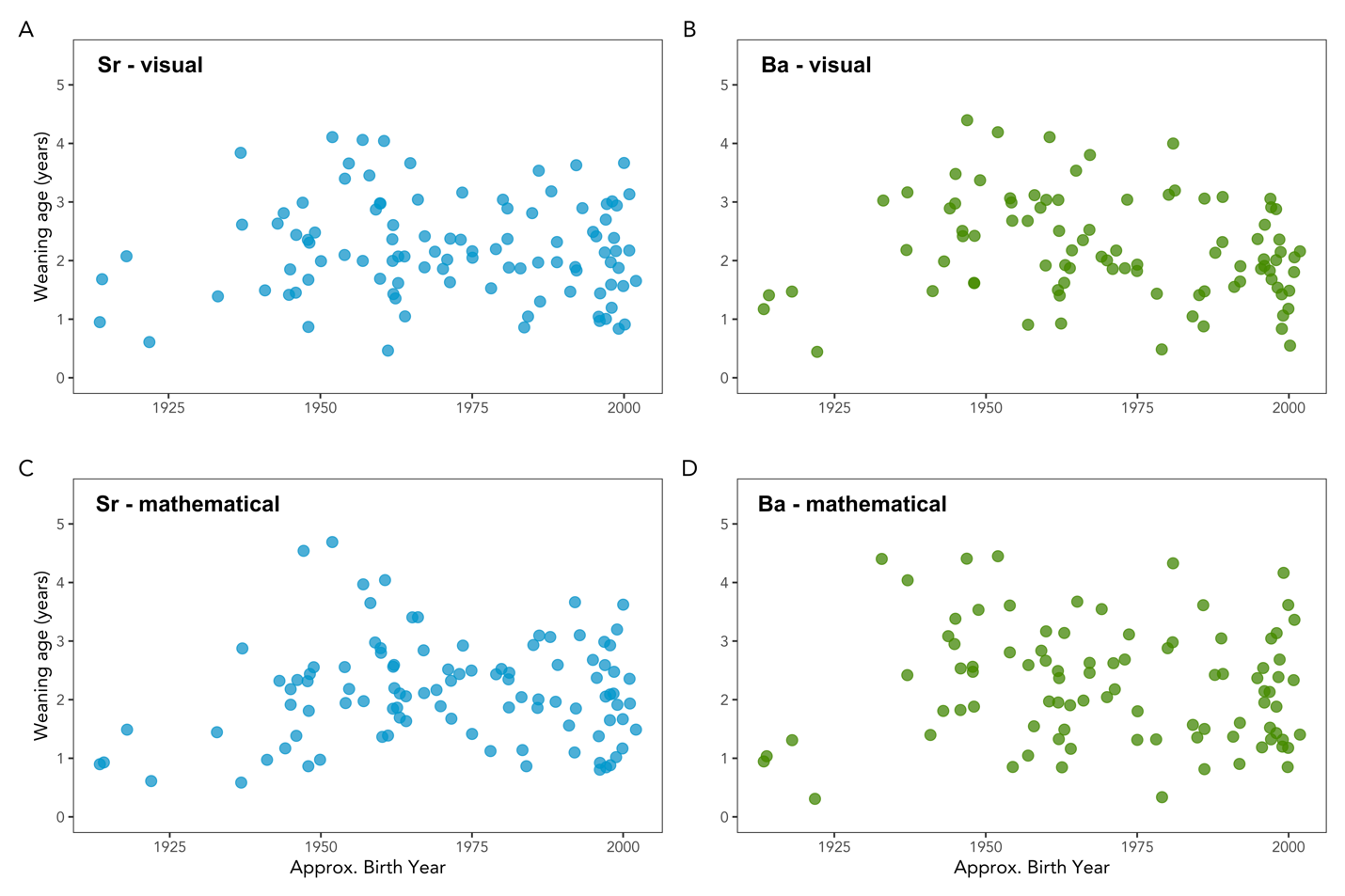


Figure S2. Hypothetical nursing and weaning timelines for walrus calves with mothers exhibiting two (top), three (middle), and four (bottom) year ovulation intervals (time between “Ovulation 1” and “Ovulation 2”). Wide, brown bars represent “maximum nursing windows” available to Calf 1, based on the assumption that Calf 1 is supplanted by Calf 2 and is unable to nurse after the birth of the second calf (Fay, 1982). Approximate timings of ovulation and birth are based on the published literature (Fay, 1982; Clark, Horstmann, & Misarti, 2020).


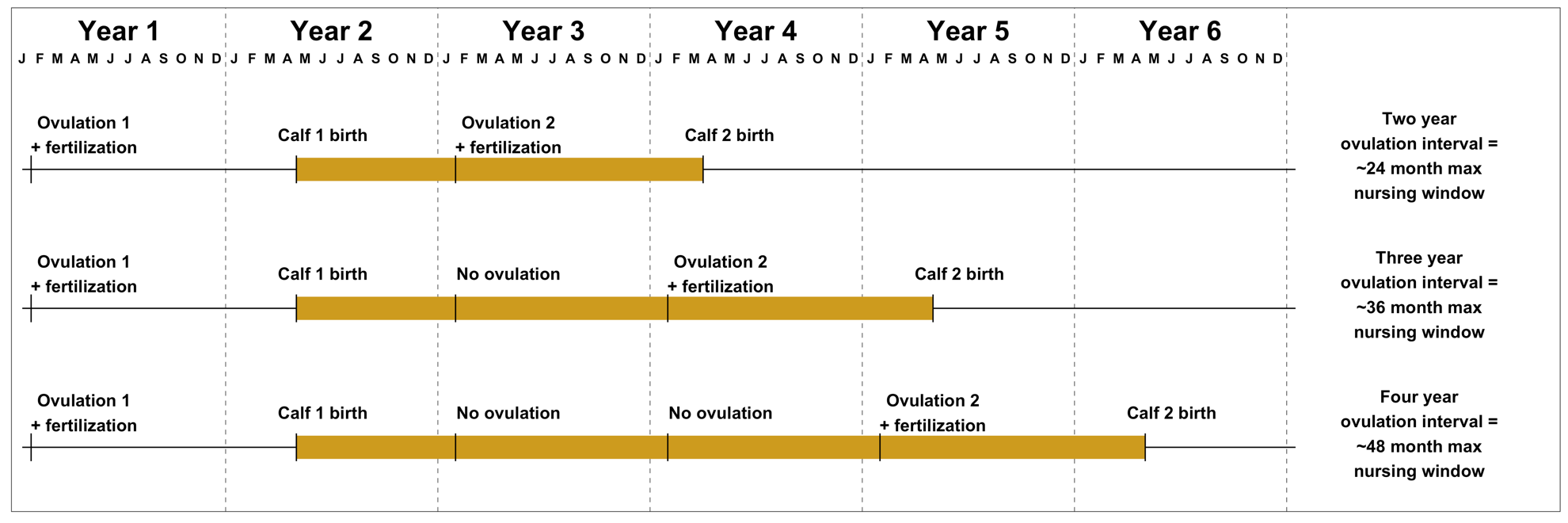


References:

Clark, C. T., Horstmann, L., & Misarti, N. (2020). Zinc concentrations in teeth of female walruses reflect the onset of reproductive maturity. *Conservation Physiology*, *8*(1), 1–13. doi:10.1093/conphys/coaa029

Fay, F. H. (1982). Ecology and biology of the Pacific walrus, *Odobenus rosmarus divergens* Illiger. *North American Fauna*, (74), 1–279. Retrieved from http://www.fwspubs.org/doi/abs/10.3996/nafa.74.0001
